# The rural pipeline to longer-term rural practice: General practitioners and specialists

**DOI:** 10.1371/journal.pone.0180394

**Published:** 2017-07-07

**Authors:** Marcella M. S. Kwan, Srinivas Kondalsamy-Chennakesavan, Geetha Ranmuthugala, Maree R. Toombs, Geoffrey C. Nicholson

**Affiliations:** 1 Rural Clinical School, The University of Queensland, Toowoomba, Queensland, Australia; 2 School of Rural Medicine, University of New England, Armidale, New South Wales, Australia; AUSTRALIA

## Abstract

**Background:**

Rural medical workforce shortage contributes to health disadvantage experienced by rural communities worldwide. This study aimed to determine the regional results of an Australian Government sponsored national program to enhance the Australian rural medical workforce by recruiting rural background students and establishing rural clinical schools (RCS). In particular, we wished to determine predictors of graduates’ longer-term rural practice and whether the predictors differ between general practitioners (GPs) and specialists.

**Methods:**

A cross-sectional cohort study, conducted in 2012, of 729 medical graduates of The University of Queensland 2002–2011. The outcome of interest was primary place of graduates’ practice categorised as rural for at least 50% of time since graduation (‘Longer-term Rural Practice’, LTRP) among GPs and medical specialists. The main exposures were rural background (RB) or metropolitan background (MB), and attendance at a metropolitan clinical school (MCS) or the Rural Clinical School for one year (RCS-1) or two years (RCS-2).

**Results:**

Independent predictors of LTRP (odds ratio [95% confidence interval]) were RB (2.10 [1.37–3.20]), RCS-1 (2.85 [1.77–4.58]), RCS-2 (5.38 [3.15–9.20]), GP (3.40 [2.13–5.43]), and bonded scholarship (2.11 [1.19–3.76]). Compared to being single, having a metropolitan background partner was a negative predictor (0.34 [0.21–0.57]). The effects of RB and RCS were additive—compared to MB and MCS (Reference group): RB and RCS-1 (6.58[3.32–13.04]), RB and RCS-2 (10.36[4.89–21.93]). Although specialists were less likely than GPs to be in LTRP, the pattern of the effects of rural exposures was similar, although some significant differences in the effects of the duration of RCS attendance, bonded scholarships and partner’s background were apparent.

**Conclusions:**

Among both specialists and GPs, rural background and rural clinical school attendance are independent, duration-dependent, and additive, predictors of longer-term rural practice. Metropolitan-based medical schools can enhance both specialist and GP rural medical workforce by enrolling rural background medical students and providing them with long-term rural undergraduate clinical training. Policy settings to achieve optimum rural workforce outcomes may differ between specialists and GPs.

## Introduction

Nearly half the world’s population live in rural and remote areas, but only about a quarter of doctors work in these areas; an inequity associated with poorer health outcomes [1]. Worldwide, most medical schools are based in large cities and recruit educationally- and financially-advantaged medical students from such cities [2], so it is not surprising that graduates tend to practice in large metropolitan cities. Although the degree, and thus impact, of medical workforce mal-distribution is greatest in lower-income countries, higher-income countries are not immune. In colonised high-income countries such as Australia, USA and Canada, Indigenous peoples are more likely to live in a rural area, which almost certainly contributes to their increased burden of disease and death. For example, in Australia two-thirds of Aboriginal and Torres Strait peoples live in rural areas and experience a 2.6-fold fatal burden compared to non-Indigenous Australians, 70% of whom live in a major metropolitan city [3].

In the absence of any randomised experimental studies [4, 5], policy interventions to address workforce mal-distribution have been informed by observational studies. An association between having a rural background and practicing in a rural area has been recognised for many decades and studies that have adjusted for confounding have reported odds ratios (ORs) of 2.3–3.5 [6–11]. Confounders identified include being single, having a rural background partner or a bonded scholarship, age, and male gender. The association has been supported by a number of reviews and commentaries [5, 8, 12–15]. Thus, it seems likely that policies that increase the proportion of rural background students entering medical schools will enhance the rural medical workforce, although questions remain regarding the nature, duration and timing of the rural exposure and possible effect modifiers.

The quality of evidence for an association between rural undergraduate clinical training and subsequent rural practice has been judged as low[4, 5] because most studies did not adjust for confounders, for example rural background. However, some exceptions have been the University of Minnesota Medical School study[16] and a study of Ontario family physicians that showed that exposure to rural undergraduate programs were independently associated with current rural practice after adjusting for rural background and other confounders [17].

Based on evidence available in 2010, WHO developed global policy recommendations summarized as—(1) recruit students with rural backgrounds, (2) locate health professional schools outside major cities, (3) clinical rotations in rural areas, (4) curricular that reflect rural health issues and (5) continuous professional development for rural health workers [1]. The quality of the evidence for (1) was considered moderate but for all others it was low to very low. A report on the early implementation of these guidelines in selected countries and regions[18] highlighted the need for more evaluation of interventions and cross-country data sharing.

Comparison of studies is problematic because between studies and countries definitions of what constitutes *rural* are variously based on distance from major metropolitan health facilities, population of towns/cities, and population density [15, 19, 20]. Likewise definitions of what is ‘rural background’, ‘rural clinical training’ and ‘rural practice’ vary considerably [21, 22].

Over the past two to three decades the Australian Government Department of Health has funded a national program to increase the rural medical workforce. At least 25% of domestic medical school entrants are required to have a rural background, defined as having resided at least five years since beginning primary school in locations classified as ‘rural’ by the Australian Standard Geographic Classification—Remoteness Areas [23] (ASGC-RA) as RA2 (Inner Regional), RA3 (Outer Regional), RA4 (Remote) or RA5 (Very Remote). Approximately 30% of the Australian population reside in RA2-5 (rural) areas and the remainder in RA1 (Major Cities or Metropolitan areas). From the early 2000s, 17 of Australia’s 18 medical schools have been funded to establish Rural Clinical Schools (RCS) to provide rural clinical training for at least one academic year for at least 25% of domestic students [24].

The University of Queensland (UQ) School of Medicine has Australia’s largest annual graduate output. Its RCS has been operating since 2002 with teaching sites in four regional cities (130-650km from the metropolitan campus), where students complete one or two years’ clinical training. UQ School of Medicine also has eight metropolitan clinical schools (MCS). In a recently reported cross-sectional cohort study[7] involving 754 UQ graduates 2002–2011, we found that rural background (as defined above) and RCS attendance were both duration-dependent independent predictors of current practice in a rural location, and that a positive multiplicative interaction exists between these two exposures. Other independent predictors were being single, having a partner with a rural background and a bonded scholarship.

Previous studies, including our own, have had a cross-sectional outcome of current practice location as rural or non-rural. Furthermore, almost all publications have been about family or primary care physicians, known as general practitioners (GPs) in Australia, with little attention to specialists who are an essential part of equitable health care. This study aimed to determine the predictors of longer-term rural practice, for both GPs and specialists.

## Methods

### Study sample

We have previously described the cohort [7]. Briefly, details of eligible participants (UQ domestic medical graduates 2002–2011) were obtained from UQ records and the Australian Health Practitioner Regulation Agency. Potential participants were invited by email, post or telephone and sent a link to an online questionnaire, or a hard copy. The University of Queensland (UQ) Human Ethics Review Committee approved the study (Ref: 2012001171), and all participants provided written informed consent prior to the study.

### Measures

Participants completed the survey reporting on demographics, residential geographic history, partnership status, rural background of parents and partner, bonded scholarships (ie recipients are required to work in a rural area for a number of years after attaining vocational qualification), details of tertiary education and post-graduate training, and locations of primary place of practice from graduation to survey date. The outcome of interest was primary place of graduate’s practice in Australia categorised as rural (ASGC-RA2-5) for at least 50% of time since graduation (‘Longer-term Rural Practice’, LTRP). The predictor variables of interest were rural background as defined above and attendance at RCS for 1 or 2 years. Potential confounding variables evaluated include all other variables listed above and year of graduation.

### Graduate background and clinical school attended

The Australian Government’s rural workforce program includes both recruitment of rural background medical students and establishment of RCS. As the program does not mandate that students with a rural background attend RCS, school allocation at UQ is based on a combination of student preference and central randomisation to achieve the required 25%. Attendance at RCS for a second year is by preference. Thus, the cohort can be divided into six sub-groups: (1) Metropolitan background and MCS (Reference group), (2) Metropolitan background and RCS-1 year, (3) Metropolitan background and RCS-2 years, (4) Rural background and MCS, (5) Rural background and RCS-1 year, and (6) Rural background and RCS-2 years.

### Graduate vocation

Vocation was classified as **GP** or **specialist** if the graduate held a Fellowship of a relevant recognised professional College or had been accepted into a Fellowship advanced training program (recognising the important role of advanced trainees in medical workforce), or **prevocational** if recognised vocational training had not commenced.

### Analyses

For statistical analyses ASGC-RA1 was considered metropolitan and ASGC-RA2-5 rural. Those currently practicing overseas were considered metropolitan as they do not contribute to the Australian rural medical workforce. Univariate and multiple logistic regression analyses were used to identify factors predictive of LTRP. Multiple regression models had adjustments for potential confounding factors. Interactions between these determinants were evaluated and included in the final model if significant. Stata for Mac (version 14.1, SE) was used for statistical analyses (College Station, Texas, USA) and p<0.05 was considered statistically significant.

## Results

### Sample characteristics

Seven hundred and fifty four graduates completed the questionnaire (48% of those potentially contactable, equivalent to 29% of all 2002–2011 UQ domestic medical graduates). The characteristics of 729 providing information relevant to this study are shown in Table 1. Of these, 32% were rural background, 37.7% attended RCS and 23.7% satisfied the primary outcome of LTRP. Specialists were the largest vocational group (49.4%). The highest proportion that was LTRP were GPs (88/224, 39.3%).

**Table 1:** Characteristics of participants (n = 729), graduates of The University of Queensland Medical program between 2002–2011.

		Mean (Standard deviation) or n (%)			
Characteristic	n with responses	All	Specialist (n = 359)	General Practice (n = 224)	Prevocational (n = 144)
**Age (years)**	727	33.3 (5.6)	33.2 (4.2)	35.5 (6.3)	30.7 (7.1)
**Female**	729	380 (52.1%)^f^	172 (47.9%)	133 (59.4%)	73 (50.7%)
**Rural background**^a^	729	233 (32.0%)^f^	106 (29.5%)	91 (40.6%)	35 (24.3%)
**Duration of rural residence prior to Medical School**	729				
<5 years (ie Metropolitan background)		496 (68.0%)^f^	253 (70.5%)	133 (59.4%)	109 (75.7%)
5 to < 10 years		72 (9.9%)	35 (9.8%)	31 (13.8%)	6 (4.2%)
10 to <15 years		119 (16.3%)	55 (15.3%)	41 (18.3%)	23 (16.0%)
≥15 years		42 (5.8%)^f^	16 (4.5%)	19 (8.5%)	6 (4.2%)
**Regional or Remote background**	729				
Regional (ASGC-RA^b^2–3)		115 (15.8%)	56 (15.6%)	39 (17.4%)	20 (13.9%)
Remote (ASGC-RA4-5)		118 (16.2%)^f^	50 (13.9%)	52 (23.2%)	15 (10.4%)
**Parent with rural background**					
Father	725	227 (31.3%)^f^	104 (29.1%)	89 (39.7%)	33 (23.4%)
Mother	719	217 (30.2%)^f^	92 (25.9%)	86 (38.6%)	38 (27.3%)
**Single**	722	180 (24.9%)	89 (25.1%)	34 (15.4%)	57 (39.6%)
**Partner with rural background**	722	145 (20.1%)^f^	56 (15.8%)	69 (31.2%)	19 (13.2%)
**Bonded scholarship**	715	81 (11.3%)^f^	28 (7.9%)	37 (16.7%)	15 (10.8%)
**RCS**^c^ **attendance**	729				
One year		165 (22.6%)	94 (26.2%)	46 (20.5%)	25 (17.4%)
Two years		110 (15.1%)^f^	45 (12.5%)	47 (21.0%)	17 (11.8%)
None (MCS^d^)		454 (62.3%)^f^	220 (61.3%)	131 (58.5%)	102 (70.8%)
**Background and RCS attendance**	729				
Metropolitan background and MCS		340 (46.6%)^f^	162 (45.1%)	92 (41.1%)	85 (59.0%)
Rural background and MCS		114 (15.6%)	58 (16.2%)	39 (17.4%)	17 (11.8%)
Metropolitan background and RCS		156 (21.4%)	91 (25.4%)	41 (18.3%)	24 (16.7%)
Rural background and RCS		119 (16.3%)^f^	48 (13.4%)	52 (23.2%)	18 (12.5%)
**Graduated from 2007**	729	444 (60.9%)^f^	199 (55.4%)	104 (46.4%)	139 (96.5%)
**Current clinical practice location**	729				
ASGC-RA1 (Metropolitan)		515 (70.7%)^f^	284 (79.1%)	126 (56.3%)	104 (72.2%)
ASGC-RA2 (Inner Regional)		102 (14.0%)^f^	31 (8.6%)	50 (22.3%)	20 (13.9%)
ASGC-RA3 (Outer Regional)		75 (10.3%)	32 (8.9%)	27 (12.1%)	16 (11.1%)
ASGC-RA4-5 (Remote/Very Remote)		23 (3.1%)	4 (1.1%)	17 (7.6%)	2 (1.4%)
Overseas		14 (1.9%)	8 (2.2%)	4 (1.8%)	2 (1.4%)
**Longer-term rural practice**^e^	727	172 (23.7%)	56 (15.6%)	88 (39.3%)	28 (19.4%)

^a^ Resided at least five years since beginning primary school in locations classified as rural, prior to commencing medical school.

^b^ASGC-RA, Australian Standard Geographical Classification-Remoteness Area

^c^Rural Clinical School.

^d^Metropolitan Clinical School

^**e**^ Rural practice ≥50% of time since graduation.

^f^ Two graduates had missing information on postgraduate status hence only appear in the All column

### Predictors of longer-term rural practice

#### Unadjusted analysis

**Fig 1:**
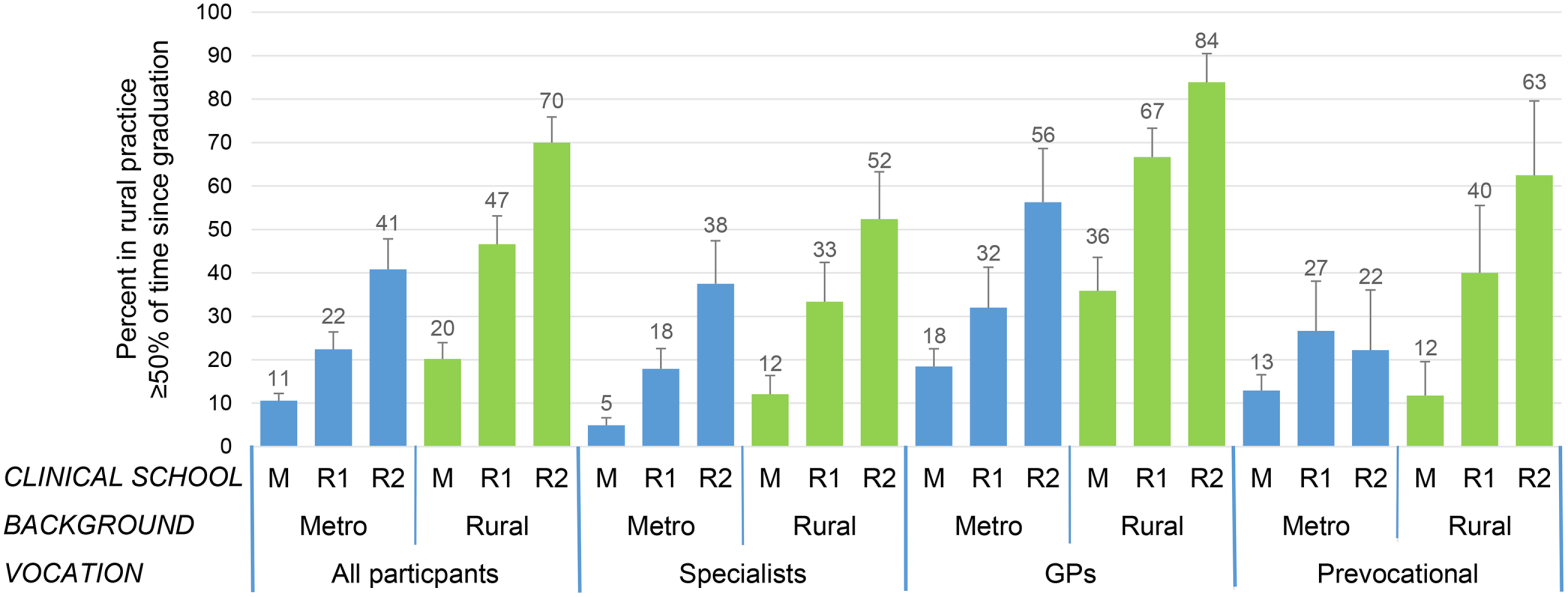
Proportion of graduates practicing in a rural area at least 50% of time since graduation. M, Metropolitan Clinical School; R1 Rural Clinical School– 1 year; R2 Rural Clinical School– 2 years; Metro, metropolitan.

Exposures associated with LTRP are shown in Table 2. The crude proportions in the six subgroups who were LTRP are shown in Fig 1. Among the reference group only 10.6% were LTRP– 5% of specialists, 18% of GPs and 13% of prevocational. However, whether metropolitan or rural background, attendance at RCS for one or two years was associated with a stepwise, approximate doubling, of the proportion who were LTRP. In those with rural background who attended RCS for two years the proportion reached 70%—specialists 52%, GPs 84% and prevocational 63%.

**Table 2:** Association of exposures with rural practice ≥50% of time since graduation.

						Vocation											
		All				Specialists				General Practice				Prevocational			
Parameter		OR	95%CI		*p*	OR	95%CI		*p*	OR	95%CI		*p*	OR	95%CI		*p*
**Age, each year increment**		**1.06**	1.03	1.09	*<0*.*001*	**0.96**	0.89	1.04	*0*.*29*	**1.07**	1.02	1.12	*0*.*005*	**1.06**	1.00	1.12	*0*.*05*
**Female**		**0.88**	0.63	1.24	*0*.*47*	**0.86**	0.48	1.52	*0*.*59*	**0.57**	0.33	0.99	*0*.*04*	**1.15**	0.50	2.64	*0*.*74*
**Rural background**^a^		**3.45**	2.42	4.93	*<0*.*001*	**2.64**	1.47	4.73	*0*.*001*	**4.25**	2.40	7.53	*<0*.*001*	**2.48**	1.03	5.99	*0*.*04*
**Rural birth place**		**2.11**	1.48	3.01	*<0*.*001*	**2.01**	1.11	3.63	*0*.*02*	**2.84**	1.59	5.05	*<0*.*001*	**1.19**	0.50	2.82	*0*.*70*
**Parent with rural background**																	
-Father		**2.96**	2.08	4.23	*<0*.*001*	**2.17**	1.22	3.87	*0*.*009*	**3.86**	2.19	6.82	*<0*.*001*	**1.14**	0.88	1.47	*0*.*33*
-Mother		**3.00**	2.09	4.29	*<0*.*001*	**1.21**	0.97	1.49	*0*.*09*	**3.17**	1.81	5.58	*<0*.*001*	**1.16**	0.95	1.42	*0*.*15*
**Partnership status**																	
-Partnered		**Ref**^b^				**Ref**				**Ref**				**Ref**			
-Single		**1.30**	0.89	1.91	*0*.*18*	**2.32**	1.27	4.24	*0*.*006*	**0.83**	0.39	1.78	0.64	**2.03**	0.88	4.68	*0*.*10*
**Partner's background**																	
-Single		**Ref**				**Ref**				**Ref**				**Ref**			
-Metropolitan		**0.38**	0.24	0.59	*<0*.*001*	**0.34**	0.16	0.71	*0*.*004*	**0.49**	0.19	1.25	*0*.*14*	**0.26**	0.08	0.79	*0*.*02*
-Rural		**2.64**	1.66	4.19	*<0*.*001*	**0.95**	0.41	2.20	*0*.*91*	**2.20**	0.81	6.00	*0*.*12*	**1.59**	0.48	5.25	*0*.*45*
**Bonded scholarship**		**4.52**	2.80	7.28	*<0*.*001*	**1.92**	0.78	4.77	*0*.*16*	**7.87**	3.39	18.28	*<0*.*001*	**3.27**	1.05	10.17	*0*.*04*
**Clinical School**																	
-MCS^c^		**Ref**				**Ref**				**Ref**				**Ref**			
-RCS^d^ – 1 or 2 years		**4.74**	3.30	6.82	*<0*.*001*	**5.72**	3.02	10.83	*<0*.*001*	**5.11**	2.86	9.12	*<0*.*001*	**3.80**	1.61	8.97	*0*.*002*
-RCS– 1 year		**3.00**	1.95	4.60	*<0*.*001*	**3.93**	1.92	8.03	*<0*.*001*	**2.96**	1.46	5.98	*0*.*003*	**3.22**	1.16	8.95	*0*.*03*
-RCS– 2 years		**8.97**	5.63	14.31	*<0*.*001*	**10.93**	4.97	24.04	*<0*.*001*	**9.41**	4.36	20.31	*<0*.*001*	**4.79**	1.55	14.80	*0*.*006*
**Background**	**Clinical School**																
Metropolitan	MCS	**Ref**				**Ref**				**Ref**				**Ref**			
	RCS-1 year	**2.44**	1.38	4.32	*0*.*002*	**4.20**	1.63	10.82	*0*.*003*	**2.08**	0.77	5.60	*0*.*15*	**2.45**	0.66	9.05	*0*.*18*
	RCS-2 years	**5.82**	2.99	11.34	*<0*.*001*	**11.55**	3.88	34.35	*<0*.*001*	**5.67**	1.85	17.37	*0*.*002*	**1.92**	0.35	10.46	*0*.*45*
Rural^a^	MCS	**2.13**	1.20	3.79	*0*.*01*	**2.64**	0.91	7.65	*0*.*07*	**2.47**	1.07	5.72	*0*.*04*	**0.90**	0.18	4.47	*0*.*89*
	RCS-1 year	**7.35**	3.95	13.68	*<0*.*001*	**9.63**	3.30	28.07	*<0*.*001*	**8.82**	3.09	25.19	*<0*.*001*	**4.48**	1.09	18.46	*0*.*04*
	RCS-2 years	**20.17**	10.53	38.63	*<0*.*001*	**21.18**	6.96	64.44	*<0*.*001*	**22.94**	7.70	68.39	*<0*.*001*	**11.21**	2.34	53.64	*0*.*002*
**Vocation**																	
-Specialist		**Ref**															
-Family/General Practice		**3.50**	2.37	5.18	*<0*.*001*												
-Prevocational		**1.31**	0.79	2.16	*0*.*30*												

^a^ Resided at least five years since beginning primary school in locations classified as rural, prior to commencing medical school.

^b^ Reference group.

^c^ Metropolitan Clinical School.

^d^ Rural Clinical School.

In general, positive associations between LTRP and the various rural exposures were similar in both specialists and GPs. However, some differences were present—Female gender (negative) and bonded scholarship were associated with LTRP in GPs, whereas being single and having a metropolitan background partner (negative) were associated in specialists.

#### Adjusted analysis

In the multivariate model with main effects that included all graduates and adjusted for background of partner, bonded scholarship and vocation (Table 3, upper section), independent predictors of LTRP were rural background, RCS attendance for 1 or 2 years, having a bonded scholarship and GP (*versus* specialist) vocation. Having a partner with a metropolitan background was a negative predictor. Other rural exposures (rural background parents, rural birthplace) exhibited multiple co-linearity with personal rural background and were not included in the model.

**Table 3:** Multivariate logistic regression models predicting rural practice ≥50% of time since graduation.

		All participants				Specialists				General Practice				Prevocational			
		OR	95% CI		*p*	OR	95% CI		*p*	OR	95% CI		*p*	OR	95% CI		*p*
**Model with Main Effects**																	
**Rural background**^a^		**2.10**	1.37	3.20	0.001	**2.10**	1.09	4.05	*0*.*03*	**2.55**	1.28	5.08	*0*.*008*	**1.61**	0.54	4.86	*0*.*40*
**Clinical School attended**																	
- MCS^c^		**Ref**^b^				**Ref**				**Ref**				**Ref**			
- RCS^d^ - 1yr		**2.85**	1.77	4.58	<0.001	**3.44**	1.62	7.28	*0*.*001*	**2.47**	1.11	5.50	*0*.*03*	**3.08**	1.01	9.34	*0*.*048*
- RCS - 2yrs		**5.38**	3.15	9.20	<0.001	**8.42**	3.60	19.69	*<0*.*001*	**4.90**	1.98	12.12	*0*.*001*	**2.83**	0.81	9.93	*0*.*10*
**Partner**																	
- No		**Ref**				**Ref**				**Ref**				**Ref**			
- Metropolitan background		**0.34**	0.21	0.57	<0.001	**0.34**	0.16	0.71	*0*.*004*	**0.49**	0.19	1.25	*0*.*14*	**0.26**	0.08	0.79	*0*.*02*
- Rural background		**1.33**	0.77	2.30	0.30	**0.95**	0.41	2.20	*0*.*91*	**2.20**	0.81	6.00	*0*.*12*	**1.59**	0.48	5.25	*0*.*45*
**Bonded scholarship**		**2.11**	1.19	3.76	0.01	**0.78**	0.26	2.34	*0*.*66*	**5.76**	2.21	14.99	*<0*.*001*	**1.54**	0.37	6.31	*0*.*55*
**Vocation**																	
- Specialist		**Ref**															
- Family/General Practice		**3.44**	2.16	5.47	<0.001												
- Prevocational		**1.39**	0.78	2.48	0.26												
**Model with interaction between background and clinical school**																	
**Background**	**Clinical School**																
Metropolitan	MCS	**Ref**				**Ref**				**Ref**				**Ref**			
	RCS-1 yr	**2.36**	1.28	4.38	*0*.*006*	**3.25**	1.23	8.57	*0*.*02*	**1.83**	0.61	5.56	*0*.*28*	**2.24**	0.56	8.87	*0*.*25*
	RCS-2 yrs	**5.09**	2.50	10.37	*<0*.*001*	**8.61**	2.79	26.5	*<0*.*001*	**6.93**	2.01	23.9	*0*.*002*	**1.32**	0.23	7.69	*0*.*76*
Rural	MCS	**1.74**	0.93	3.26	*0*.*08*	**2.00**	0.65	6.23	*0*.*23*	**2.49**	0.98	6.36	*0*.*06*	**0.71**	0.13	4.00	*0*.*70*
	RCS-1 yr	**6.58**	3.32	13.04	*<0*.*001*	**7.52**	2.50	22.6	*<0*.*001*	**8.75**	2.71	28.3	*<0*.*001*	**5.33**	1.02	27.7	*0*.*047*
	RCS-2 yrs	**10.36**	4.89	21.93	*<0*.*001*	**16.5**	5.06	53.9	*<0*.*001*	**8.50**	2.50	28.9	*0*.*001*	**7.20**	1.12	46.3	*0*.*04*
**Partner**																	
**-** No partner		**Ref**				**Ref**				**Ref**				**Ref**			
- Metro background		**0.34**	0.21	0.57	*<0*.*001*	**0.34**	0.16	0.72	*0*.*005*	**0.46**	0.18	1.20	*0*.*11*	**0.24**	0.08	0.75	*0*.*02*
- Rural background		**1.35**	0.78	2.34	*0*.*28*	**0.96**	0.42	2.22	*0*.*93*	**2.20**	0.80	6.04	*0*.*13*	**1.64**	0.47	5.74	*0*.*44*
**Bonded scholarship**		**2.12**	1.19	3.79	*0*.*01*	**0.79**	0.26	2.35	*0*.*67*	**6.22**	2.36	16.4	*<0*.*001*	**1.39**	0.30	6.51	*0*.*67*
**Vocation**																	
- Specialist		**Ref**															
- Family/General Practice		**3.40**	2.13	5.43	*<0*.*001*												
- Prevocational		**1.37**	0.77	2.45	*0*.*29*												

^a^ Resided at least five years since beginning primary school in locations classified as rural prior to commencing medical school.

^b^ Reference group.

^c^ Metropolitan Clinical School

^d^ Rural Clinical School

After stratification by vocation, rural background and RCS attendance remained predictors of LTRP in both specialists and GPs. However, having a metropolitan-background partner was not a significant negative predictor in GPs and having a bonded scholarship was not a significant positive predictor in specialists.

#### Interaction between background and clinical school

The multivariate model that included all three vocational groups and an interaction term of background and clinical school attended showed that, compared to the reference group, attendance at RCS for one or two years was a significant predictor of LTRP regardless of background (Table 3, lower panel). However, MCS attendance mitigated the effect of rural background.

Among specialists, interaction between background and clinical school exhibited a pattern similar to that seen in the GPs or the whole cohort. Among GPs, a duration-depend effect of RCS was not present and the rural background effect tended to persist despite MCS attendance. The effect of a bonded scholarship was only apparent in GPs, whereas the negative effect of having a metropolitan partner was not significant in GPs.

#### Interaction between duration of rural background and clinical school

**Fig 2:**
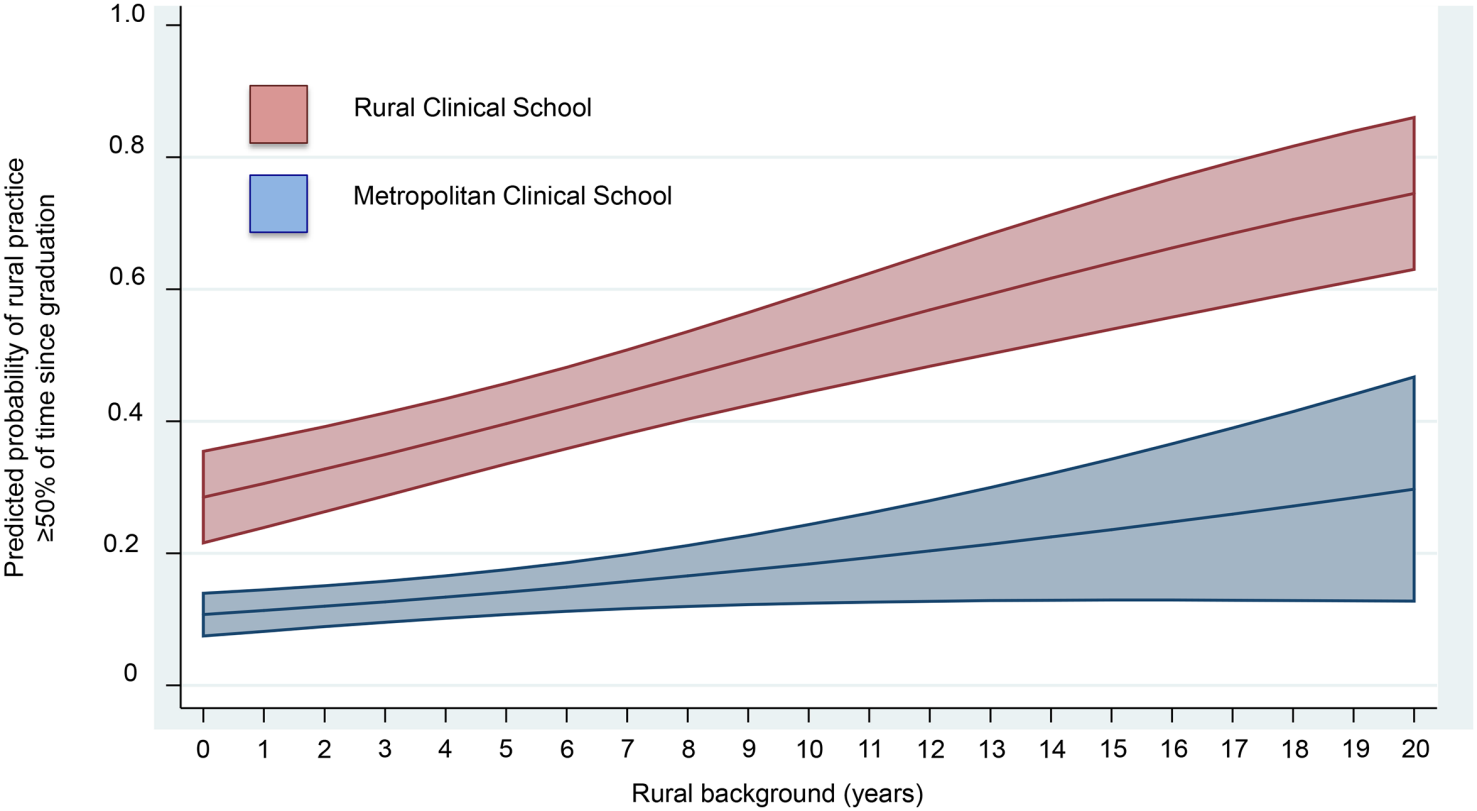
Adjusted predications of rural practice by years of rural background and Clinical School attended. The logistic regression model included RCS *versus* MCS, years resided in a rural location prior to medical school as a continuous variable, and an interaction between these two variables. Rural background—years resided in a rural area (ASGC-RA 2–5) prior to entering medical school. The shaded areas are 95% confidence intervals.

To examine the effect of the duration of rural background on the adjusted predictive probability of LTRP amongst all participants, we developed a logistic regression model with explanatory variables: RCS *versus* MCS, years resided in a rural location prior to medical school as a continuous variable, and an interaction between these two variables (Fig 2). The predicted probabilities are divergent across 0 to 20 years duration of rural background. In RCS attendees with 10 and 20 years of rural background, the predicted probabilities (95% CI) of rural practice are 52% (44–60%) and 75% (64–86%), respectively.

## Discussion

### Key results

This study shows that rural background and one or two years RCS attendance are strong independent predictors of LTRP in both GPs and specialists. Rural background and RCS attendance additively interact to increase the probability of rural practice—the combination of rural background and two years of RCS attendance is associated with the highest probability of rural practice– 84% in GPs and 52% in specialists.

### Models of rural clinical training

A long-standing approach to rural doctor shortage in the USA and Canada has been the establishment of comprehensive medical rural programs that include various combinations of recruitment of rural background or rural-committed students, and required rural curricula or rural clinical training for six months or longer.[10, 25, 26] A systematic review that included ten studies reporting such programs found that the weighted average of graduates in rural practice ranged from 53–64% depending on the definition of rural [20]. The UQ RCS program, which is arguably ‘comprehensive’ because 40% of students are rural background and all received a year or more rural clinical training, has achieved similar outcomes: Amongst those with a rural background who attended RCS for one year and two years 46.6% and 70.5%, respectively, are in LTRP.

Another successful model is that of regional/rural medical schools where the main campus is located in a rural area [2, 27]. Examples include Memorial University of Newfoundland Medical School based in St Johns (population 100,000)[2] and the Northern Ontario School of Medicine (NOSM) based in Sudbury (population 160,000) [27]. Both enrol high proportions of rural background students, provide rural undergraduate clinical training and a high proportion of their graduates are in rural family practice. An Australian example is James Cook University located in far north Queensland. A recent analysis[28] of 229 James Cook University medical graduates 2005–2008, reported that independent predictors of rural practice in their fifth postgraduate year were rural hometown, general practice, location of internship and Indigenous heritage. Another model, generically termed ‘Rural Longitudinal Integrated Clerkships’, involves placing students, often with a rural background, in rural community primary care settings for extended periods, has been employed in many medical schools in multiple countries [29].

### Specialists *versus* general practitioners

An association between rural background and rural specialist practice was previously identified using the dataset of a prospective cohort study of Australian doctors [30]. Among 2425 specialists (22.2% with a rural background), who were unrestricted in their workplace location, childhood residence in a rural area of 11–18 years duration was associated with current rural practice after adjusting for gender and age group (OR 2.27 95% Confidence Interval (CI) 1.77–2.91). In 3156 GPs, the association was seen with a shorter rural residence of 6–10 years (OR 2.28 95% CI 1.69–3.08). No adjustments for other covariates such as rural clinical school attendance, partnership status or bonded scholarship were made. This study supports our finding that rural background predicts rural practice among specialists. The effect of rural undergraduate clinical training on rural specialist practice has not previously been adequately studied.

Australian Government strategies to increase the rural general practice workforce, including rural postgraduate training programs and rural incentives, together with an almost doubling of the number of medical graduates over the past 15 years, have resulted in a significant increase the rural GP numbers. In 2001 the full time equivalent (FTE) rate (per 100,000 population, based on a 45 hour week) for GPs in Australia was lower in rural compared to metropolitan areas—FTE rates were 106, 91, 87, 89 and 92 for RA1, RA2, RA3, RA4 and RA5, respectively. Among specialists the decline with increasing rurality was more marked– 115, 54, 33, 19 and 5 respectively [31, 32]. By 2012, GP FTE rates were higher in rural than metropolitan areas– 108, 118, 123, 134 respectively for RA1, RA2, RA3 and RA4/5. In contrast, among specialists the sharp decline in specialist FTE rates with increasing rurality persisted –153, 79, 58 and 33, respectively [32].

The paucity of both public and private specialist employed positions in rural areas is probably a major factor contributing to low specialist FTE rates outside of major cities. This in turn, together with the metropolitan-centric approaches of the specialist Colleges has resulted in a paucity of specialist training positions. In 2012, of 11,478 employed Specialists-in-training, only 13.8% were resident outside RA1. In stark contrast, 32.5% of 4908 GP vocational trainees were training through the rural pathway [33].

Of the 30% of Australians who do not live in major cities, the vast majority (27.5%) live in regional areas (ASGC-RA2-3), mostly in larger cities and towns, whereas only 2.3% live in remote areas. In 2009–2011, 86% of the potentially avoidable deaths in Australia related to not living in major cities (n = 18,954) occurred in regional areas and only 14% in remote areas [34]. In countries with widely dispersed populations it is not feasible to provide metropolitan-equivalent health services to the entire non-metropolitan population. Strategies that close health services gaps in regional areas will be the most effective and feasible way to reduce inequity in health experienced by rural populations.

Primary care has an essential role in health systems[35] and is critically important to rural communities and in areas of low population density where GPs are the only resident doctors. Thus it is appropriate for GP rates to increase as the population density and specialist rates decrease. Nevertheless, the current situation where specialist rates are half to a third in regional areas compared to the metropolitan cities is likely a significant contributor to the excess of avoidable deaths outside of major cities. The situation is similar in the USA and Canada [15, 36]. Unlike the super-specialisation that has developed in major cities, larger regional centres are best served by generalist specialists in surgery, internal medicine, anaesthetics, psychiatry, paediatrics and obstetrics. In the past, quintessential rural GPs provided both primary care and secondary specialist care in smaller rural and regional hospitals but their numbers have declined in the last few decades largely due to credentialing and indemnity insurance issues. Recently, they have been successfully reinvented in Australia, Canada and elsewhere as the ‘rural generalist’ [37, 38]. Rural Generalists are trained in rural primary care and also have enhanced skills (and credentialing) in one or more specialties, such as emergency medicine, obstetrics and gynecology, anesthetics, surgery and others. Essentially, rural generalists are both rural GPs and rural specialists.

### Add-on benefits of training medical students in rural areas

In addition to increasing the likelihood that graduates will enter longer-term rural practice, RCS and rural medical schools can build rural workforce and expertise by recruiting experienced clinical academics (GPs and specialists) to teach and provide clinical services. They also enhance rural communities (and thus workforce retention) by providing research and continuing medical education opportunities, reducing the isolation of local clinicians, and providing employment and training opportunities for local people. By leveraging the existing resources of the main metropolitan medical school, RCS are likely to be less expensive to establish and maintain than stand-alone regional or rural medical schools.

### Limitations and strengths

A potential limitation of our study is that intention to practice in a rural area and its role in influencing location of clinical placement during medical school (ie RCS *versus* MCS) is not known. Students entering their first year of clinical training (year three of the medical course) are able to preference their clinical schools in rank order. Computerised matching is done centrally and when a Clinical School is over-subscribed with first preferences, the matching proceeds to second preferences and so on. The result is that students who preference RCS, and those who do not, can be allocated to an RCS. Since our study is retrospective and the Australian Government did not mandate the proportion allocated to RCS during the study period, the numbers in each category are not available. Likewise the “rural intention” of our graduate cohort is not available. Recent confidential surveys done on entry to UQ RCS in year three, however, suggest that rural intent is not the main driver for choosing a RCS placement. When asked ‘What was the main reason for choosing the clinical school that you are currently attending?’ with one of six possible responses allowed, 52% of 61 students entering RCS nominated ‘reputation of the clinical school’ and 23% ‘subsidised accommodation’. Only 4.9% nominated ‘I chose RCS because I want to eventually practice in a rural location’. There was no significant difference between the proportion of MCS and RCS students who nominated the latter (p = 0.107).

The study cohort represents only 29.4% of 2478 UQ domestic medical graduates 2002 to 2011. However, as previously described [7], apart from a higher proportion of RCS attendees, the characteristics of this cohort are similar to those of 2360 UQ medical graduates 2002–2011, making selection bias unlikely. With 38.5% of the population living in RA2-5 areas, Queensland is the most ruralised of Australia’s major states, allowing increased opportunity for rural practice. Consequently, our results may not be generalizable to other Australian states or international sites with lower rural population proportions. Furthermore, although the program is national, universities are permitted to apply the program rules differently so each RCS has its own enrolment criteria, curriculum and course structure. With this variability, together with the significant geographical and rural population differences that exist both within and between states, outcomes would be expected to be different at each RCS.

Results in the pre-vocation group should be interpreted in the light of the smaller number in this group, their significantly younger age, and the fact that their work locations are largely controlled by factors beyond their control such as state health department allocations to hospital residencies and post-graduate training positions.

Although not a randomised controlled study, our study reports a natural experiment[39] with an intervention group (RCS) and a control group (MCS), both completing the same curriculum at different sites. The data are from a large number of graduates over a decade and sufficient numbers have been exposed to both rural background and RCS, and belong to the main vocational groups of specialist and GP. We acknowledge that unmeasured or unknown confounders may have influenced the outcomes of our study.

### Policy implications

The weight of current evidence is that although rural background or rural undergraduate clinical training individually increase the probability of rural practice in both specialists and general practitioners, it is the combination of these two exposures that provides a ‘rural pipeline’ and best predicts this outcome. This is true whether the model is a comprehensive medical rural program, regional/rural medical school, integrated longitudinal clerkships or a metropolitan medical school with a RCS. Since the supply of rural background medical students and rural undergraduate clinical training opportunities will always be limited, the most efficient way to use these limited resources to enhance the rural medical workforce, both GP and specialist, is to combine them by facilitating RCS attendance for rural background students. Mandating RCS attendance for students who gain entry to the medical school through the rural background quota scheme could be considered.

Our results suggest that strategies to increase the rural medical workforce may differ according to whether GP or specialist. The associations between rural background, RCS and rural practice are remarkably similar in specialists and GPs, yet the crude proportion in LTRP is much less among specialists than GPs (15.6% vs 39.3%). In the adjusted analysis GPs were 3.5-fold (95% CI 2.37, 5.18) more likely than specialists to be in LTRP. Among modifiable exposures, the proportion with a rural background is marginally lower (29.5% vs 40.6%) and the proportion that attended RCS is similar (38.7% vs 41.5%). The proportion attending RCS for two years, however, is substantially lower among specialists (12.5% vs 21.0%), which was likely to reduce the LTRP proportion. We have no data regarding reasons why such students do only one year but a possible factor is that they are keen to return to the metropolitan clinical schools to “position” themselves for a specialist training position, the vast majority of which are metropolitan. Thus, strategies that increase the number of specialist training positions in rural areas are likely to increase the number of rural specialists in a number of ways—by reducing the incentive for students planning to be specialists to return to metropolitan clinical schools or do internships at metropolitan hospitals, and by allowing them complete basic and advanced specialist vocational training in rural areas—ie constructing a rural pipeline for specialists. In contrast, a third of GP vocational training positions are now rural so the GP rural pipeline is operational. Having a bonded rural scholarship is associated with a 7.87-fold probability of GP LTRP suggesting that these schemes should be boosted. Among GPs with a metropolitan background, those who attended RCS for one year do not have an increased probability of LTRP compared to those who did not attend RCS. This outcome suggests that these RCS positions should be preferentially offered to students with a rural background although such schemes create equality issues. Furthermore, students may not decide on their vocation until after graduation.
